# Supplementary material for: Short-term effects of thinning on the development and communities of understory vegetation of Chinese fir plantations in Southeastern China
Source: PeerJ. 2020 Feb 11;8:e8536. doi: 10.7717/peerj.8536 (PMC7020821; doi:10.7717/peerj.8536)
Supplement: Table S1 [file peerj-08-8536-s001.docx]

**Table S1:**

**Importance value of understory plants on different thinning intensities.**

| Stand  layers | Family | Genus | Species | Thinning intensity | | | |
| --- | --- | --- | --- | --- | --- | --- | --- |
|  |  |  |  | CK | LIT | MIT | HIT |
| Shrub layers | Verbenaceae | *Callicarpa* | *C. giraldii* | 0.229 | 0.452 | 0.368 | 0.383 |
|  |  | *Premna* | *P. microphylla* |  | 0.037 | 0.056 | 0.005 |
|  | Moraceae | *Ficus* | *F. hirta* | 0.348 | 0.111 | 0.166 | 0.109 |
|  | Myrsinaceae | *Maesa* | *M. perlarius* | 0.082 | 0.092 | 0.032 | 0.097 |
|  |  | *Ardisia* | *A. lindleyana* | 0.064 | 0.107 | 0.075 | 0.052 |
|  | Rubiaceae | *Mussaenda* | *M. pubescens* | 0.014 | 0.085 | 0.032 | 0.097 |
|  | Symplocaceae | *Symplocos* | *S. sumuntia* | 0.112 | 0.029 |  | 0.005 |
|  |  |  | *S. lancifolia* | 0.021 |  | 0.018 | 0.017 |
|  | Ericaceae | *Vaccinium* | *V. carlesii* | 0.061 | 0.010 |  |  |
|  | Sabiaceae | *Meliosma* | *M. rigida* | 0.023 |  | 0.037 |  |
|  | Lauraceae | *Litsea* | *L. cubeba* |  |  |  | 0.016 |
|  |  | *Machilus* | *M. grijsii* |  | 0.030 | 0.013 | 0.007 |
|  | Sapindaceae | *Cardiospermum* | *C. halicacabum* |  |  | 0.016 | 0.034 |
|  | Berberidaceae | *Nandina* | *N. domestica* |  |  | 0.032 | 0.012 |
|  | Leguminosae | *Millettia* | *M. speciosa* | 0.047 |  | 0.024 | 0.009 |
|  |  |  | *M. nidita* |  |  | 0.030 |  |
|  |  |  | *M. dielsiana* |  |  | 0.007 | 0.033 |
|  | Magnoliaceae | *Kadsura* | *K. longipedunculata* |  | 0.037 |  |  |
|  | Hamamelidaceae | *Loropetalum* | *L. chinense* |  |  | 0.021 | 0.016 |
|  | Theaceae | *Eurya* | *E. nitida* |  |  | 0.031 |  |
|  |  |  | *E. rubiginosa* var. *attenuata* |  |  |  | 0.018 |
|  | Rosaceae | *Rubus* | *R. lambertianus* |  |  | 0.024 | 0.016 |
|  |  |  | *R. corchorifolius* |  |  |  | 0.023 |
|  |  |  | *R. alceifolius* |  |  | 0.006 |  |
|  |  |  | *R. rosifolius* |  |  |  | 0.013 |
|  | Aquifoliaceae | *Ilex* | *I. purpurea* |  |  |  | 0.013 |
|  | Saxifragaceae | *Ribes* | *R. fasciculatum* |  |  | 0.007 | 0.006 |
|  | Araliaceae | *Aralia* | *A. elata* |  |  |  | 0.011 |
|  | Euphorbiaceae | *Antidesma* | *A. pseudomicrophyllum* |  | 0.011 |  |  |
|  |  | *Sapium* | *S. discolor* |  |  | 0.006 |  |
|  |  | *Glochidion* | *G. puberum* |  |  |  | 0.005 |
| Herb layers | Athyriaceae | *Allantodia* | *A. metteniana* | 0.267 | 0.165 | 0.176 | 0.108 |
|  |  |  | *A. virescens* |  | 0.027 | 0.036 | 0.016 |
|  |  | *Acystopteris* | *A. japonica* | 0.051 | 0.062 | 0.081 | 0.099 |
|  | Blechnaceae | *Woodwardia* | *W. japonica* | 0.181 | 0.115 | 0.088 | 0.100 |
|  |  | *Blechnum* | *B. orientale* | 0.039 | 0.070 | 0.097 | 0.072 |
|  | Thelypteridaceae | *Dictyocline* | *D. wilfordii* | 0.123 | 0.101 | 0.084 | 0.086 |
|  |  | *Parathelypteris* | *P. glanduligera* |  | 0.010 | 0.007 | 0.031 |
|  | Dennstaedtiaceae | *Microlepia* | *M. hancei* | 0.120 | 0.077 | 0.066 | 0.074 |
|  | Lindsaeaceae | *Odontosoria* | *O. chinensis* | 0.022 | 0.086 | 0.124 | 0.075 |
|  | Gramineae | *Lophatherum* | *L. gracile* | 0.075 | 0.037 |  | 0.092 |
|  |  | *Setaria* | *S. viridis* |  |  |  | 0.014 |
|  | Liliaceae | *Ophiopogon* | *O. bodinieri* | 0.006 | 0.048 | 0.060 | 0.048 |
|  | Adiantaceae | *Adiantum* | *A. flabellulatum* | 0.012 | 0.075 | 0.024 | 0.012 |
|  |  |  | *A. juxtapositum* | 0.009 | 0.004 |  |  |
|  | Dryopteridaceae | *Cyrtomium* | *C. conforme* | 0.028 | 0.048 | 0.026 | 0.019 |
|  |  | *Dryopteris* | *D. fuscipes* | 0.014 | 0.007 | 0.024 | 0.027 |
|  |  |  | *D. labordei* |  | 0.010 |  |  |
|  |  |  | *D. erythrosora* |  |  | 0.006 |  |
|  |  | *Arachniodes* | *A. amoena* |  |  | 0.005 | 0.003 |
|  |  |  | *A. coniifolia* |  |  | 0.003 |  |
|  | Pteridaceae | *Pteris* | *P. semipinnata* | 0.012 | 0.028 | 0.025 | 0.030 |
|  | Gleicheniaceae | *Dicranopteris* | *D. pedata* | 0.023 | 0.009 | 0.028 | 0.026 |
|  | Lygodiaceae | *Lygodium* | *L. japonicum* | 0.017 | 0.015 | 0.018 | 0.032 |
|  | Commelinaceae | *Commelina* | *C. communis* |  |  | 0.006 | 0.015 |
|  | Angiopteridaceae | *Angiopteris* | *A. fokiensis* |  | 0.007 | 0.012 |  |
|  | Rosaceae | *Duchesnea* | *D. indica* |  |  |  | 0.012 |
|  | Selaginellaceae | *Selaginella* | *S. tamariscina* |  |  |  | 0.007 |
|  | Rubiaceae | *Paederia* | *P. scandens* |  |  |  | 0.003 |
|  | Melastomataceae | *Melastoma* | *M. dodecandrum* |  |  | 0.003 |  |
